# Use of Organosilicon Compounds towards the Rational Design of Antiparasitic and Antiviral Drugs

**DOI:** 10.1155/MBD.1995.143

**Published:** 1995

**Authors:** Gérard Déléris

**Affiliations:** 1 Bioorganic Chemistry Laboratory University of Bordeaux 2 Bordeaux F-33076 France

## Abstract

One of the major problems met for the conception of antiviral or antiparasitic drugs is to reach a high level of selectivity towards the pathogenic agent versus the host. We shall describe two synthetic approaches where main group organometallics have been used towards this goal. A series of nucleoside sila-analogues was synthesized as potential therapeutic agents designed to inhibit HIV Reverse Transcriptase. In a second approach novel organosilicon derivatives have been synthesized as mimics of antisense oligonucleotides.

Infectious agents, namely viruses or parasites, more or less use cellular machinery. Therefore therapeutic agents must interfere with biochemical mechanisms or possess high affinity towards specific molecular cellular components, to reach selectivity.

We thought that main group organometallics could show many advantages for designing biologically active molecules in this field. They allow a high synthetic flexibility for the modulations of physico-chemical properties and they show a mechanistic behaviour which may be close to the one of several heteroelements present in living organisms such as sulfur or phosphorus.

We tried to use this approach towards two directions involving the synthesis of organosilicon derivatives i.e:

-the synthesis of organosilicon derivatives as inhibitors of HIV Reverse Transcriptase,

-the synthesis of organosilicon precursors of modified antisense oligonucleotides.


### USE OF ORGANOSILICON COMPOUNDS TOWARDS THE RATIONAL DESIGN OF ANTIPARASITIC AND ANTIVIRAL DRUGS

Gérard Déléris
Bioorganic Chemistry Laboratory, University of Bordeaux 2, F-33076 Bordeaux France

**Abstract:** One of the major problems met for the conception of antiviral or antiparasitic drugs is to reach a high level of selectivity towards the pathogenic agent versus the host. We shall describe two synthetic approaches where main group organometallics have been used towards this goal. A series of nucleoside sila-analogues was synthesized as potential therapeutic agents designed to inhibit HIV Reverse Transcriptase. In a second approach novel organosilicon derivatives have been synthesized as mimics of antisense oligonucleotides.

Infectious agents, namely viruses or parasites, more or less use cellular machinery. Therefore therapeutic agents must interfere with biochemical mechanisms or possess high affinity towards specific molecular cellular components, to reach selectivity.

We thought that main group organometallics could show many advantages for designing biologically active molecules in this field. They allow a high synthetic flexibility for the modulations of physico-chemical properties and they show a mechanistic behaviour which may be close to the one of several heteroelements present in living organisms such as sulfur or phosphorus.

We tried to use this approach towards two directions involving the synthesis of organosilicon derivatives i.e:

-the synthesis of organosilicon derivatives as inhibitors of HIV Reverse Transcriptase,

-the synthesis of organosilicon precursors of modified antisense oligonucleotides.

### 1- Organosilicon Derivatives as Potential Inhibitors of HIV Reverse Transcriptase.

This enzyme is one of the most important targets (1) for the development of antiretrovirals against HIV. It catalyses the RNA directed synthesis of viral DNA (DNA pol activity) and in a second step the degradation of the RNA/DNA double strand (RNase H activity). Most of the RT inhibitors so far approved for clinical use, as for example AZT or $D_4T$ (2)(scheme 1), are not actual RT inhibitors but DNA chain elongation terminators. Other classes of inhibitors have been developped, as HEPT or TIBO and others (3). These ones are no longer DNA chain elongation inhibitors but they interact with a non catalytic site within the enzyme.

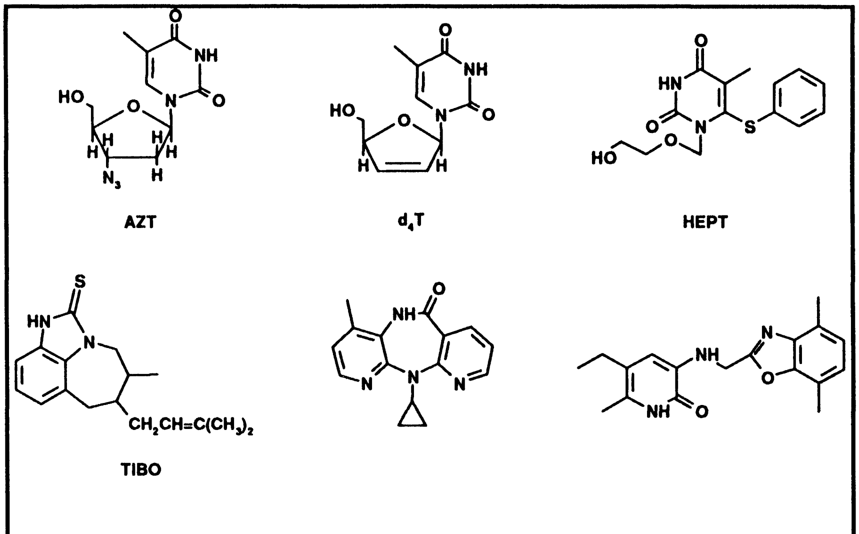

Scheme 1

✱ Conference given in ICEBAMO 94 (International Conference on Environmental and Biological Aspects of Main-Group Organometals, Bordeaux 06-09/09/94).

Thus any mutation of this protein lowers their affinity without affecting catalytic potency.

We tried to imagine structures of potential inhibitors from the biochemical mechanism of DNA polymerase which is shown on scheme 2. On this scheme is shown the nucleophilic attack of the last grafted nucleotide on the α-phosphorus of the nucleotide which will be next linked, with expulsion of the diphosphate moiety which is in apical position.

So we decided to synthesize either monosubstrate- or disubstrate-analogues, examples of which are shown on scheme 3a,3b.

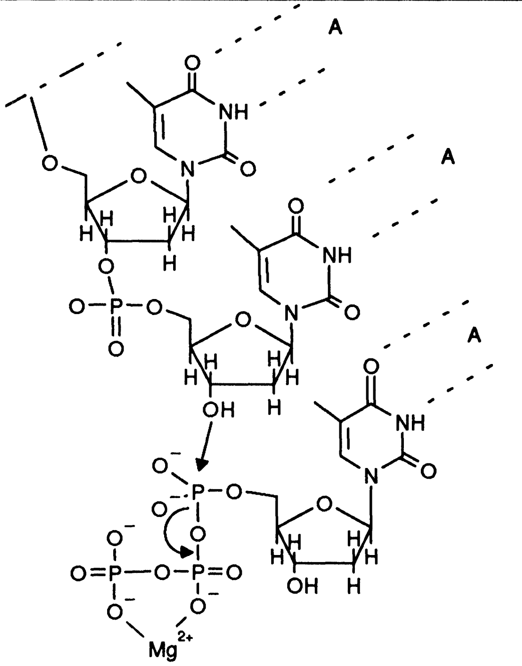

Scheme 2

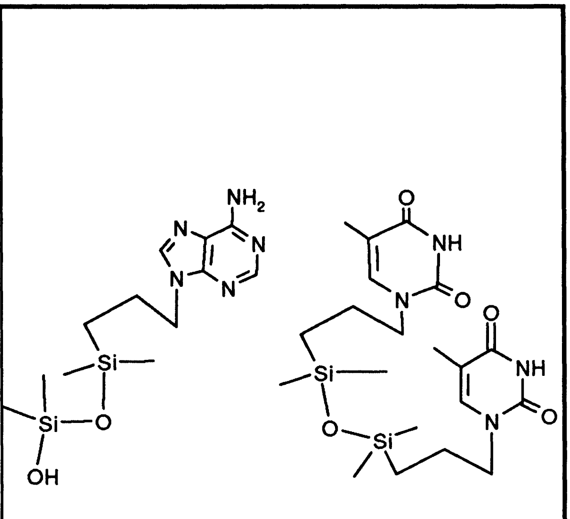

Scheme 3

The synthesis of monosubstrate analogues was performed with thymine and adenine as bases. It required an hydrosilylation reaction (4) of the allyl base as shown on scheme 4. Yields were low, 12% for thymine and 25% for adenine, due to difficulties encountered for compound purification.

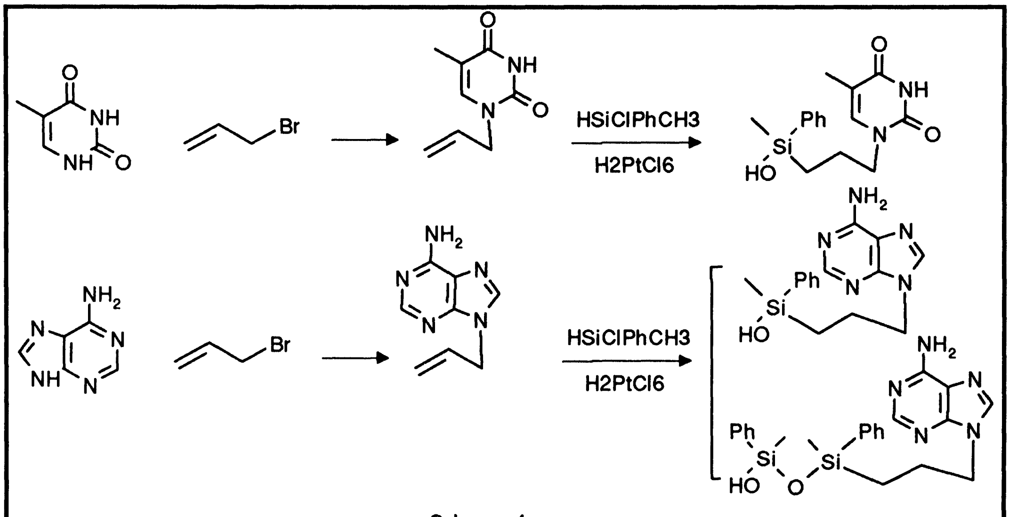

Scheme 4

Bisubstrate analogues was synthesized following two ways.

The first one is shown on scheme 5. It requires the condensation of thymine, adenine or of an heterocyclic sodium salt on chloropropyl-dimethyl chlorosilane followed by hydrolysis (or on the corresponding disiloxane). Results are gathered in table 1. For thymine and adenine we could obtain mixtures or regio-isomers which were separated.

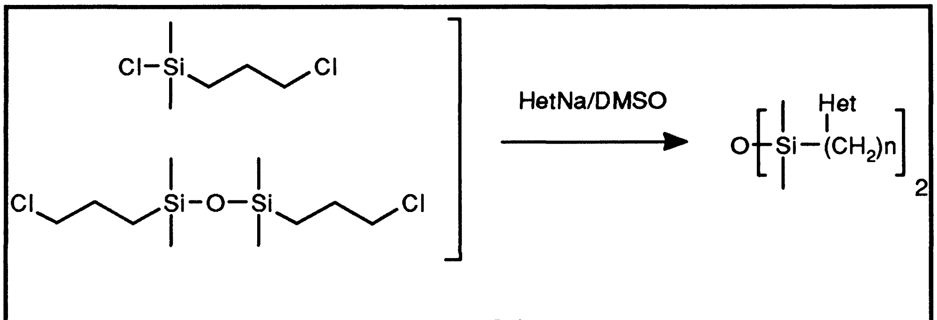

Scheme 5

| Heterocycle | Products | Yield (%) |
|---|---|---|
|  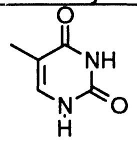 |  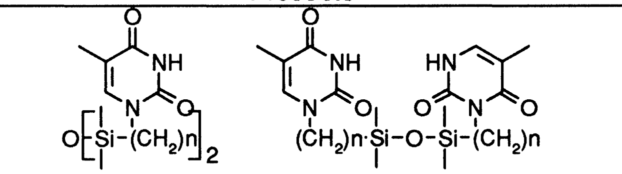 | 18 |
|  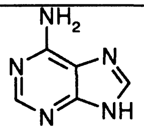 |  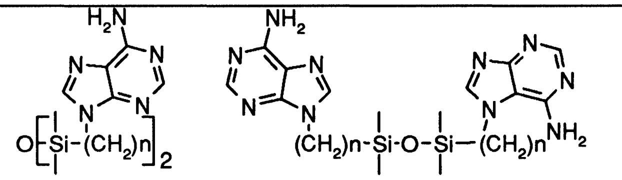 | 40 |
|  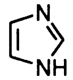 |  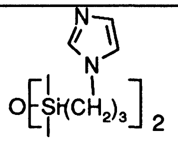 | 59 |
|  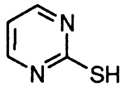 |  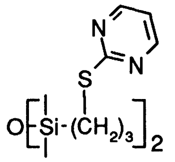 | 47 |
|  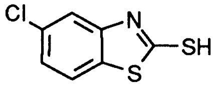 |  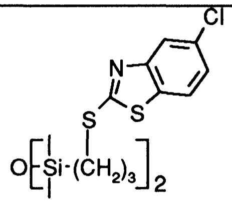 | 21 |
|  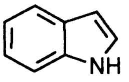 |  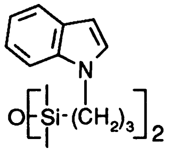 | 27 |

**Table 1**

The second synthetic way was acylation reaction of bis-aminopropyltetramethyldisiloxane with isatoic anhydrides from which we obtained orthoamino benzamides (Yields 65-95%). These ones were then cyclized and reduced to quinazoline derivatives (Yields ca 60%)(scheme 6).

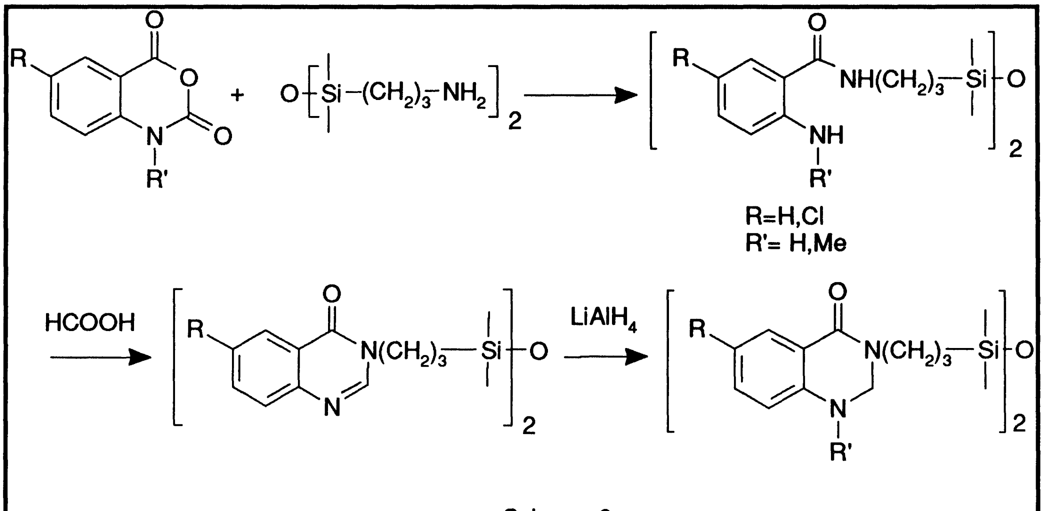

Scheme 6

The synthesized compounds were tested in IBGC CNRS Bordeaux (Dr S.Litvak ), for their activity as inhibitors of recombinent HIV RT, with poly-rA oligodT as template for catalytic activity. Most of them did not show any significant activity. Only compounds 132, 82b and 91 showed activities which ranged from $IC_{50}$=20 to 125 µm and could be compared with TIBO activity, i.e 60 µM in the same model. More accurate analyses were performed (scheme 7). RNase H activity was found to be much less affected than RNA directed-DNA polymerase activity as well by compound 132 as by compound 82B (scheme 9). In every case $IC_{50}$ inhibitory concentrations were shown to be respectively 20 and 60 µm and cellular DNA α-polymerase was not affected by these compounds till 500 µM.

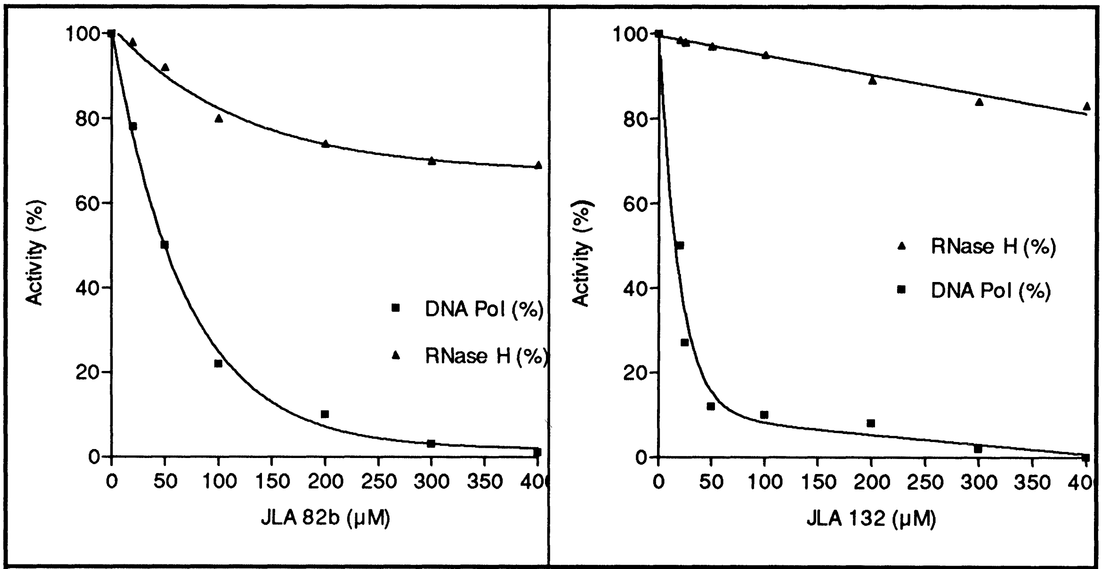

Scheme 7

The activity of the synthesized compounds has been studied on cultures of HIV-1 infected MT2 cells for their antiretroviral properties, and uninfected for their cytotoxic activities, in the

Laboratoire de Virologie Humaine, Université de Bordeaux II (Pr H. Fleury). Results, presented on scheme 8, highlight that most of these compounds show either a high cytotoxicity (low $CD_{50}$) with good antiretroviral properties or a low activity (high $ID_{50}$). Only compound 82b exhibits a good ratio between its antiretroviral activity ($ID_{50} \approx 12\ \mu M$) and its cytotoxicity on uninfected MT2 cells ($CD_{50} > 100\ \mu M$).

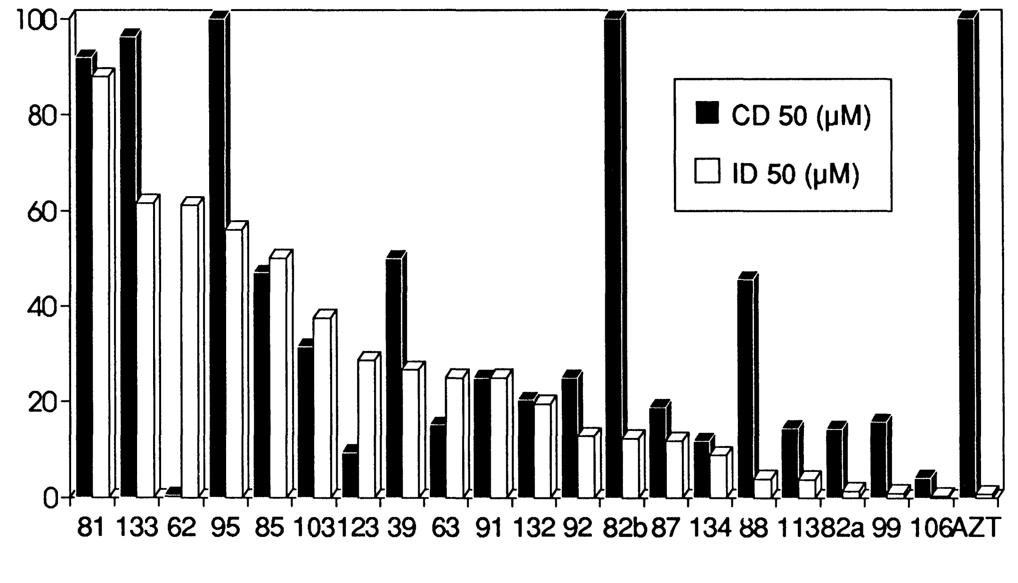

Scheme 8

As this compound had been designed not to be an AZT-like DNA elongation terminator, it was studied for its antiretroviral properties on AZT-susceptible (BGL) and AZT-resistent (BRG) clinical isolates of HIV-1 obtained from a patient prior to and following initiation of AZT therapy. Close levels of activity were observed for both strains ($IC_{50} \approx 13\ \mu M$, Scheme 9).

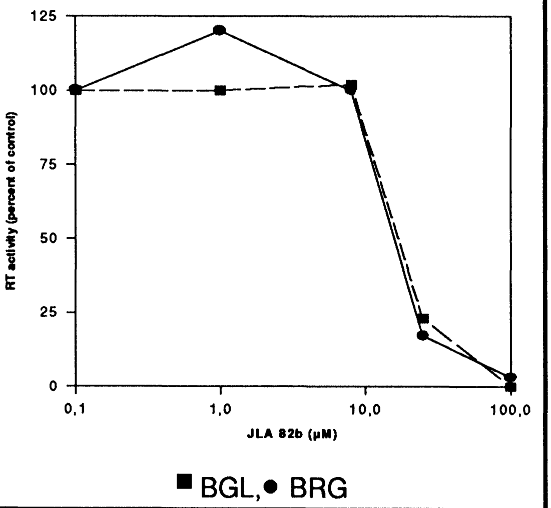

Scheme 9

### 2- Organosilicon precursors of modified antisense oligonucleotides.

Antisense approach deals(5,6) with the synthesis of oligonucleotides complementary of either DNA or RNA sequences. This complementarity, which confers a very high selectivity to the base-pairing, allows to block either transcription if the target is DNA or translation if the target is m-RNA (7,8). Thus one has to synthesize oligonucleotides the length of which most often vary from 13 to 20 nucleotides (9).

These antisense nucleotides, if not modified, suffer from several limitations:

-they undergo rapid enzymatic degradation by nucleases,

-they offer a poor cellular penetration,

-they only afford a narrow range for the modulation of their physico-chemical properties

-they present a lot of difficulties arising from deoxyribose chemistry (10).

In order to overcome these difficulties, modifications have been introduced within the structure of antisense oligonucleotides, mainly dealing with the modification of the phosphodiester linkage, synthesis of phosphorothioates, or phosphonates (1 and 2 Scheme 10).

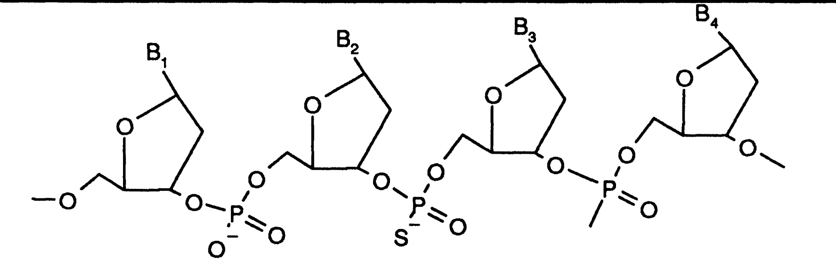

1 2

Scheme 10

We underwent the synthesis of organosilicon precursors of modified oligonucleotides. The chemical target was chosen (shown on scheme 11) such as ribose is replaced by a linear chain which bears two hydroxy functions in pseudo-3' and pseudo-5' positions, and a silicon atom on the pseudo-4' position.

Silicon atom is linked to one chain which embodies a three carbon linker and a nucleic base. The fourth valence remaining on silicon is variable and could allow modulations of the physico-chemical properties of the molecule.

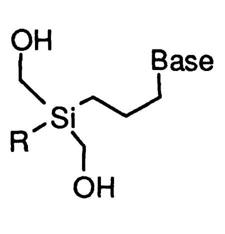

Scheme 11

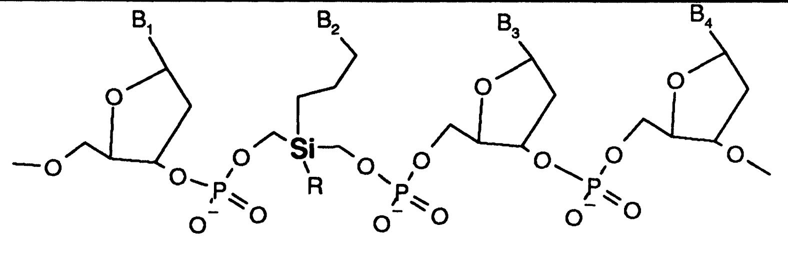

Scheme 12

The final goal is the insertion of this monomeric unit within an oligonucleotide in order to obtain antisense oligodeoxynucleotides (Scheme 12).

The synthesis of the monomers was performed with thymine and adenine as the nucleic bases. Hydrosilylation of allyl-substituted bases being the key step for introducing a silicon atom on the structure, hydrogenosilanes were first synthesized by reduction of the corresponding chlorosilane as shown on scheme 13.

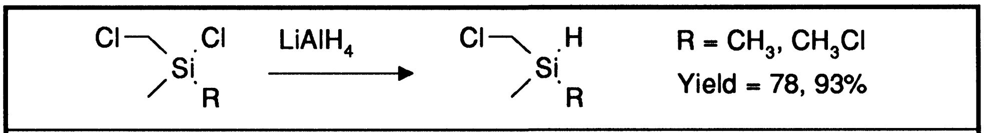

Scheme 13

Then was performed the synthesis of the thymine monomer as shown on scheme 14. The process required allylation of bis-trimethylsilyl protected thymine, followed by hydrosilylation, acetoxylation of the chloromethyl moiety, and subsequent saponification which yielded respectively alcohol or diol ( overall yield 5% and 13%).

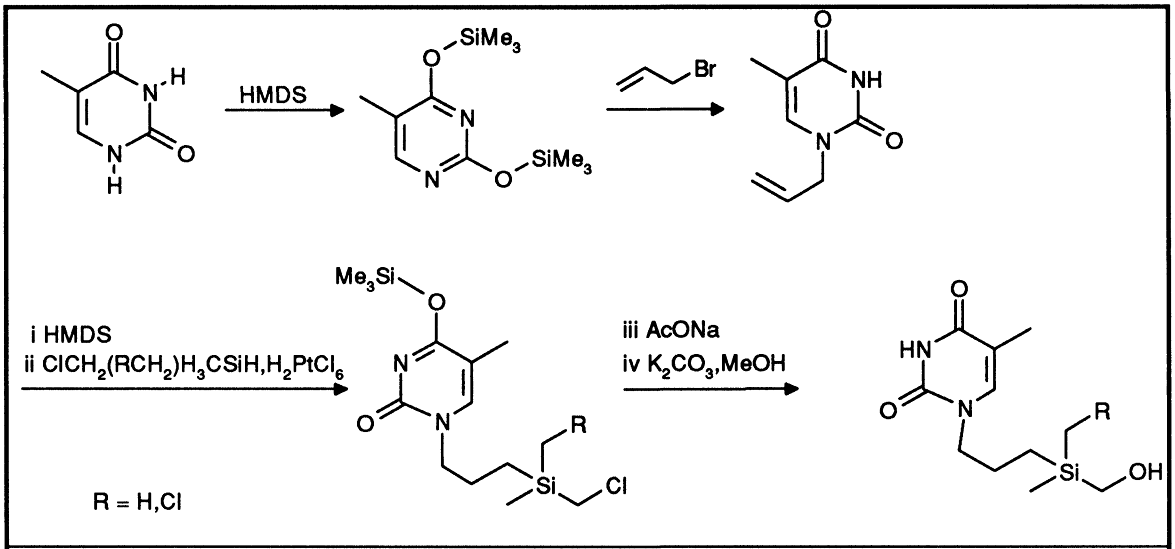

Scheme 14

As hydrosilylation, when performed on allyl-adenine, gave very poor yields (ca 3%), even after protection, we started from the readily available 6-chloropurine (Scheme 15). Its sodium salt was allylated with allyl bromide (65%).

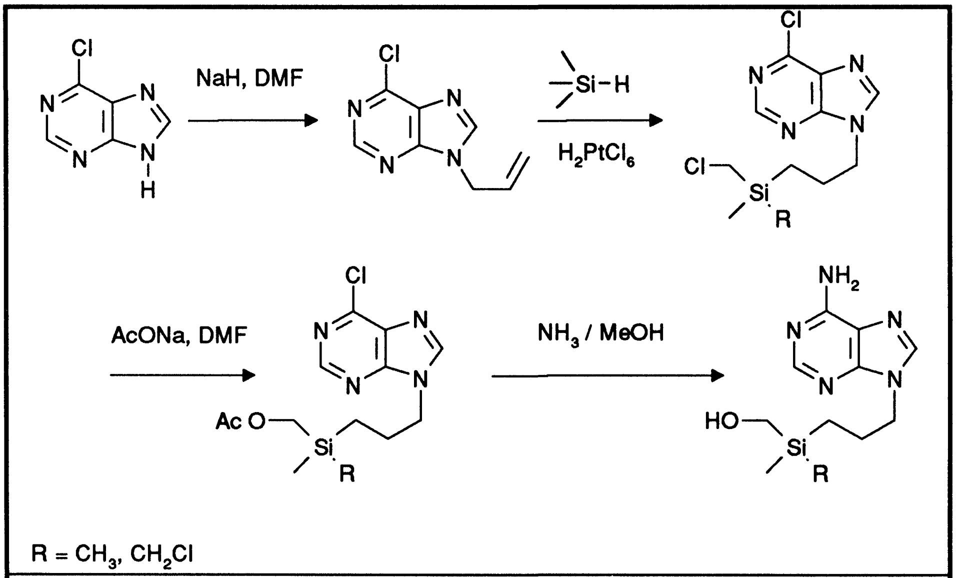

Scheme 15

Subsequent hydrosilylation, yielded organosilicon derivatives (yield= 57% R= $CH_2Cl$and 17% R=Me). Then acetoxylation (66%) and reaction with methanolic ammonia (65% and 50%) yielded desired adenyl derivatives .

Thymine monomer was protected as 4,4'-dimethoxytrityl ether and then reacted with commercial diisopropyl,β–cyanoethoxy chlorophosphoramidite, as shown on scheme 16, in collaboration, by Dr S. Moreau (INSERM U386, Bordeaux).

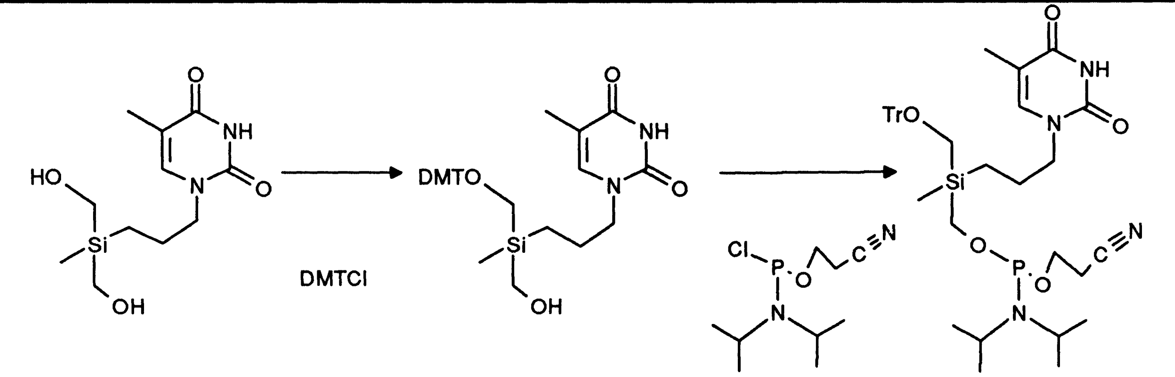

Scheme 16

So was obtained a sila-analogue of a nucleotide including both protective and activating groups which allow its use in solid phase oligonucleotide synthesis on an automatic synthesizer. This monomer was incorporated within a 17mer deoxyoligonucleotide complementary of a part of the coding sequence for rabbit β–globin:

**5'-TT GTG $^{Si}$T CA AAA GCA AGT-3'.**

Studies to determine the influence of this substitution on the physico-chemical properties of base pairing (Tm) are currently underway.

**Acknowledgments**

I would like to warmly acknowledge participations to this work of my coworkers, Jean Lafay, Laurent Latxague, Jacques Thibon, Christel Guillot, and those of Laura Litvak (Laboratoire de Réplication des Rétrovirus IBGC CNRS, S. Litvak Dir.), Brigitte Delord (Laboratoire de Virologie Humaine, H. Fleury Dir.) and Serge Moreau (Laboratoire de Biophysique Moléculaire INSERM U386, J.J. Toulmé Dir.). Moreover financial support from Conseil Régional d'Aquitaine and A.R.C. is kindly acknowledged.

**References**

1 (a) Fischl, M.A.; Richman, D.D.; Grieco, M. H. *N. Engl. J. Med.* 1987, **317**, 185-191. (b) De Clercq, E. *J. Acquired Immune Defic. Syndr.* 1991, **4**, 207-218. (c) Yarchoan, R.; Broder, S. *Pharmacol. Ther.* 1989, **40**, 329-384. (d) Yarchoan, R.; Mitsuya, H.; Thomas, R.V.; Pluda, J.M.; Hartman, N.R.; Perno, C.F.; Marczyk, K.S.; Allain J.P.; Johns, D.G.; Broder, S. *Science* 1989, **245**, 412-415.

2 (a) Richman, D.D.; Fischl, M.A.; Grieco, M.H.; Gottlieb, M.S.; Volberding, P.A.; Laskin, O.L.; Leedom, J.M.; Groopman, J.E.; Mildvan, D.; Hirsch, M.S.; Jackson, G.G.; Durack, D.T.; Nusinoff-Lehrman, S. *N. Engl. J. Med.* 1987, **317**, 192-197. (b) Yarchoan, R.; Pluda, J.M.; Thomas, R.V.; Mitsuya, H.; Brouwers, P.; Wyvill, K.M.; Hartman, N.; Johns, D.G.; Broder, S. *Lancet* 1990, **336**, 526-529.

3 (a) Tanaka, H.; Baba, M.; Hayakawa, H.; Sasamaki, T.; Mizayaka, T.; Ubasawa, M.; Takashima, H.; Sekiya, K.; Nitta, I.; Shigeta, S.; Walker, R. T.; Balzarini, J.; De Clercq, E., *J. Med. Chem.*, 1991, **34**, 349.

(b) Kukla, M. J.; Breslin, H. J.; Pauwels, R.; Fedde, C. L.; Miranda, M.; Scott, M. K.; Sherrill, R. G.; Raeymaekers, A.; Van Gelder, J.; Andries, K.; Janssen, M. A. J.; De Clerq, E.; Janssen, P. A. J., *J. Med. Chem.*, 1991, **34**, 746.
(c) Merluzzi, V. J.; Hargrave, K. D.; Labadia, L. et al., *Science*, 1990, **250**, 1411.
(d) Goldman, M. E.; Nunberg, J. H.; O'Brien, J. A.; Quintero, J. C.; Schleif, W. A.; Freund, K. F.; Gaul, S. L.; Saari, W. S.; Wai, J. S.; Hoffman, J. M.; Anderson, P. S.; Hupe, D. J.; Emini, E. A.; Stern, A. M., *Proc. Natl. Acad. Sci. U.S.A.*, 1991, **88**, 6863.
(e) Romero, D. L.; Busso, M.; Tan, C. K.; Reusser, F.; Palmer, J. R.; Poppe, S. M.; Aristoff, P. A.; Downey, K. M.; So, A. G.; Resnick, L.; Tarpley, W. G., *Proc. Natl. Acad. Sci. U.S.A.*, 1991, **88**, 8806.

4 (a) Ryan, J., Menzie, G., Speier, J.L.; *J. Am. Chem. Soc.*, 1960, **82**, 3601.
(b) Weber, W., *"Silicon reagents for organic synthesis"* Springer Verlag, Berlin, 1983.

5 Dan Cook, P. *Anti-Cancer Drug Design* 1991, **6**, 585-607.

6 Hélène, C.; Toulmé, J.-J. *Biochim. Biophys. Acta* 1990, **1049**, 99-125.

7 Zamecnik, P.C.; Stephenson, M.L. *Proc. Natl. Acad. Sci. USA* 1978, **75**, 280-284.

8 Stephenson, M.L.; Zamecnik, P.C. *Proc. Natl. Acad. Sci. USA* 1978, **75**, 285-288.

9 Uhlmann, E.; Peyman, A. *Chem. Rev.* 1990, **90**, 543-584.

10 Nielsen, P. E.; Egholm, M.; Buchardt, O. *Bioconjugate Chem.* 1994, **5**, 3-7 and references cited herein.

**Received: January 5, 1995 - Accepted: February 14, 1995 - Received in revised camera-ready format: March 14, 1995**

